# Multispectral and Molecular Docking Studies Reveal Potential Effectiveness of Antidepressant Fluoxetine by Forming π-Acceptor Complexes

**DOI:** 10.3390/molecules27185883

**Published:** 2022-09-10

**Authors:** Ahmed Gaber, Walaa F. Alsanie, Majid Alhomrani, Abdulhakeem S. Alamri, Hussain Alyami, Sonam Shakya, Hamza Habeeballah, Heba A. Alkhatabi, Raed I. Felimban, Abdulwahab Alamri, Abdulhameed Abdullah Alhabeeb, Bassem M. Raafat, Moamen S. Refat

**Affiliations:** 1 Department of Biology, College of Science, Taif University, P.O. Box 11099, Taif 21944, Saudi Arabia; 2 Centre of Biomedical Sciences Research (CBSR), Deanship of Scientific Research, Taif University, P.O. Box 11099, Taif 21944, Saudi Arabia; 3 Department of Clinical Laboratories Sciences, The Faculty of Applied Medical Sciences, Taif University, P.O. Box 11099, Taif 21944, Saudi Arabia; 4 College of Medicine, Taif University, P.O. Box 11099, Taif 21944, Saudi Arabia; 5 Department of Chemistry, Faculty of Science, Aligarh Muslim University, Aligarh 202002, India; 6 Department of Medical Laboratory Technology, Faculty of Applied Medical Sciences in Rabigh, King Abdulaziz University, P.O. Box 80203, Jeddah 21589, Saudi Arabia; 7 Department of Medical Laboratory Sciences, Faculty of Applied Medical Sciences, King Abdulaziz University, P.O. Box 80203, Jeddah 21589, Saudi Arabia; 8 Center of Excellence in Genomic Medicine Research (CEGMR), King Abdulaziz University, P.O. Box 80203, Jeddah 21589, Saudi Arabia; 9 King Fahd Medical Research Centre, Hematology Research Unit, King Abdulaziz University, P.O. Box 80203, Jeddah 21589, Saudi Arabia; 10 Center of Innovation in Personalized Medicine (CIPM), 3D Bioprinting Unit, King Abdulaziz University, P.O. Box 80203, Jeddah 21589, Saudi Arabia; 11 Department of Pharmacology and Toxicology, College of Pharmacy, University of Hail, P.O. Box 2240, Hail 55476, Saudi Arabia; 12 National Centre for Mental Health Promotion, P.O. Box 95459, Riyadh 11525, Saudi Arabia; 13 Department of Radiological Sciences, College of Applied Medical Sciences, Taif University, P.O. Box 11099, Taif 21944, Saudi Arabia; 14 Department of Chemistry, College of Science, Taif University, P.O. Box 11099, Taif 21944, Saudi Arabia

**Keywords:** fluoxetine HCl, major depressive disorder, π-acceptor complexes, molecular docking, spectroscopy

## Abstract

Poor mood, lack of pleasure, reduced focus, remorse, unpleasant thoughts, and sleep difficulties are all symptoms of depression. The only approved treatment for children and adolescents with major depressive disorder (MDD) is fluoxetine hydrochloride (FXN), a serotonin selective reuptake inhibitor antidepressant. MDD is the most common cause of disability worldwide. In the present research, picric acid (PA); dinitrobenzene; p-nitro benzoic acid; 2,6-dichloroquinone-4-chloroimide; 2,6-dibromoquinone-4-chloroimide; and 7,7′,8,8′-tetracyanoquinodimethane were used to make 1:1 FXN charge-transfer compounds in solid and liquid forms. The isolated complexes were then characterized by elemental analysis, conductivity, infrared, Raman, and ^1^H-NMR spectra, thermogravimetric analysis, scanning electron microscopy, and X-ray powder diffraction. Additionally, a molecular docking investigation was conducted on the donor moiety using FXN alone and the resulting charge transfer complex [(FXN)(PA)] as an acceptor to examine the interactions against two protein receptors (serotonin or dopamine). Interestingly, the [(FXN)(PA)] complex binds to both serotonin and dopamine more effectively than the FXN drug alone. Furthermore, [(FXN)(PA)]–serotonin had a greater binding energy than [FXN]–serotonin. Theoretical data were also generated by density functional theory simulations, which aided the molecular geometry investigation and could be beneficial to researchers in the future.

## 1. Introduction

Depression is a common mental illness that affects almost 300 million people of all ages around the world [1]. Depression’s effects can last a long time or recur, and they can have a substantial influence on a person’s ability to function and live a happy life. Major depressive disorder (MDD) is the primary cause of disability worldwide. At least one distinct depressive episode lasting at least two weeks, as well as evident changes in mood, interests, and pleasure, cognitive changes, and vegetative symptoms, characterize this severe condition. Girls are more likely than boys to be affected by MDD, with a global incidence of 3.0% and 1.8%, respectively [2].

MDD is the second leading cause of chronic illness among all medical diseases according to the years lived with disability [3]. Additionally, MDD is associated with a higher risk of contracting diseases, such as diabetes, heart disease, and stroke, which ultimately enhances the severity of the disease [4]. Notably, death by suicide can be induced by MDD. It is estimated that up to 50% of around 800,000 suicides committed annually around the world take place during a depressed episode [5], and MDD patients are approximately 20-fold more likely than the general population to commit suicide [6].

Fluoxetine hydrochloride (FXN) was first described as a selective serotonin (5-HT)-uptake inhibitor in 1974 [7] and was the initial representative of a new group of antidepressant medications identified as selective serotonin reuptake inhibitors. In fact, FXN was the first of this class to be released in the United States. Notably, inhibitors of this class are still among the most often prescribed antidepressants today [8]. Moreover, FXN is the only approved treatment for pediatric and adolescent patients with MDD [9].

Charge transfer (CT), which involves an electron donor interacting with several acceptors, is now a significant factor in the study of drug–receptor binding mechanisms [10] and several biological fields [11]. As a result, CT interactions of some acceptors have been successfully used in pharmacokinetic analysis [12], with extensive studies conducted on CT–receptor compounds for broad applications [13]. 

Chemists, biologists, and pharmacists are all interested in the chemistry of CT interactions and complex building, particularly when it comes to pharmaceuticals. Their interest in CT complexes is related to their high value and numerous uses in a variety of industries and technological, chemical, biological, and pharmaceutical fields. For instance, CT complexes have been employed in biological, electrical, optical, and magnetic experiments, as well as in investigations of pharmaceutical receptor-binding mechanisms [14,15,16]. The development of quick, dependable, and straightforward techniques for the qualitative and quantitative detection of drugs in bulk and/or pharmaceutical dose forms was also based on the production of CT complexes [17,18].

In pharmaceutical formulations, π-acceptors were employed in the spectrophotometric examination of numerous medicines [19,20,21,22,23,24,25]. Recent studies have demonstrated the potential efficacy of using π-acceptors to boost antidepressant drug levels [26,27,28]. 

In the current study, the CT complexes of FXN with six different types of π-acceptors were synthesized and thoroughly characterized at different levels in both solid and liquid forms. The interactions between two protein receptors (serotonin or dopamine) and ligands were also examined by molecular docking using Autodock Vina software. To understand how receptor–ligand interactions work, several molecular dynamic simulations were run. Multiple degrees of comparisons were made between CT complexes of FXN and the FXN drug alone against the serotonin receptor.

## 2. Results and Discussion

### 2.1. Multispectroscopic Studies

Recently, there has been a lot of focus on the development of stable CT complexes that come from the interaction of electron acceptors with chemical or biological molecules. The important physical and chemical characteristics of these complexes are the source of this interest. Compared with the methods of drug determination described previously in the literature, the CT complexation is an important approach that is less complicated, more affordable, and more effective [29]. Understanding the interactions between medications and receptors as well as the mechanisms behind drug action may be possible through the study of drug CT complexes [30].

In the present research, a molar ratio of 1:1 was used for the elemental analyses of all synthesized FXN-CT complexes, which are easily soluble in dimethylformamide and dimethylsulfoxide. The molar conductance values of the CT complexes of FXN revealed a slightly electrolytic nature (37–56 Ω^−1^ cm^−1^ mol^−1^) upon the creation of positive and negative datives anions owing to hydrogen bonding.

**Figure 1:**
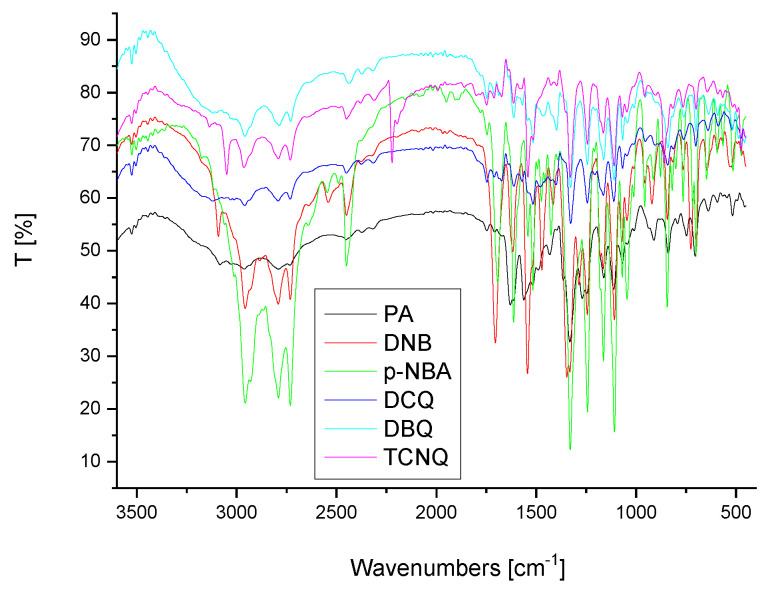
FTIR spectra analysis of FXN drug generated with six *π*-acceptors.

The infrared spectrum of FXN had different stretching vibration bands at 3500 cm^−1^ (N–H stretching), 3200–3000 cm^−1^ (C−H stretching), 1600–1400 cm^−1^ (C=C stretching), 1300 cm^−1^ (C–O stretching), and 1200–1000 cm^−1^ (C–F stretching) [31]. After CT complexation, the υ(N–H) band was absent, and the δ(NH) band shifted to lower wavenumbers (1611–1629 cm^−1^). These alterations could result from a single pair of -NH electrons interacting with the six acceptors. The *n*–π* CT transitions caused the C=C stretching frequency to decrease from 1590 cm^−1^ to 1569–1537 cm^−1^. In addition, the stretching vibration of υ(C–N) shifted to 1349–1324 cm^−1^ in all six acceptors with the FXN drug complexes according to the interaction between –NH and the acceptors (Figure 1).

**Figure 2:**
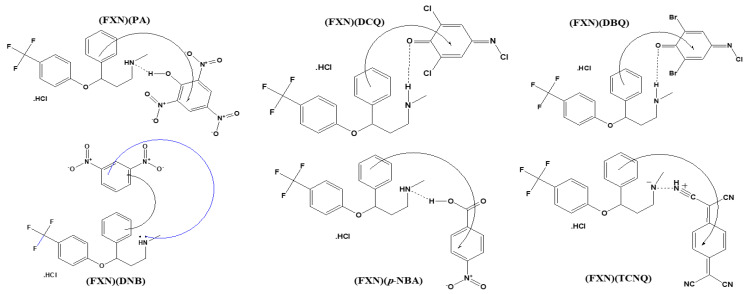
Molecular structures between FXN and the six π-acceptors.

Few bands were observed from 2373–2452 cm^−1^ due to intermolecular hydrogen bonding between the basic nitrogen of FXN as an *n*-donor (D) and the *π*-acceptor (A), resulting in CT complexes of the *n*-*π* type (D–A) (Figure 2) [32].

**Figure 3:**
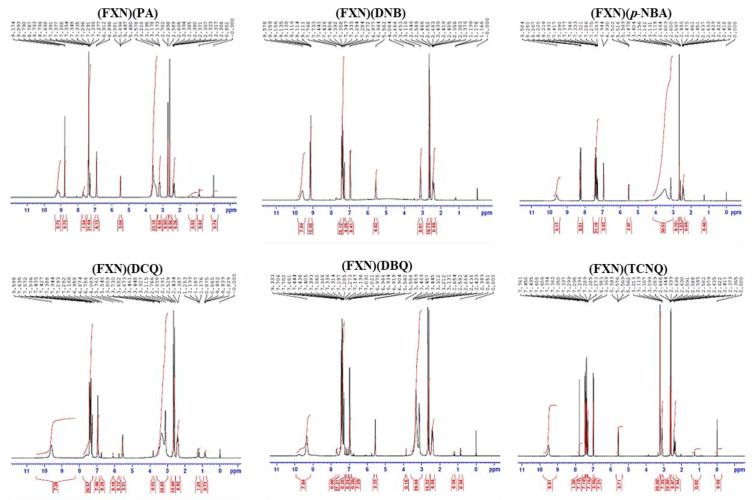
^1^HNMR spectra of the six ligand complexes.

^1^HNMR spectra of the FXN with the six π-acceptors complexes are shown in Figure 3. The –NH peak of the secondary amine of FXN shifted to a lower field (9.11–9.58 ppm) due to the involvement of the -NH proton on the receptor fragment (FXN). Aromatic protons also shifted downfield in the spectra of the FXN complexes. Such a result strengthens the transfer of electrons from the lone electron pair on the nitrogen atom of the secondary amine group –NH to the acceptors and aromatic rings of the FXN donor via intermolecular hydrogen bonding.

**Figure 4:**
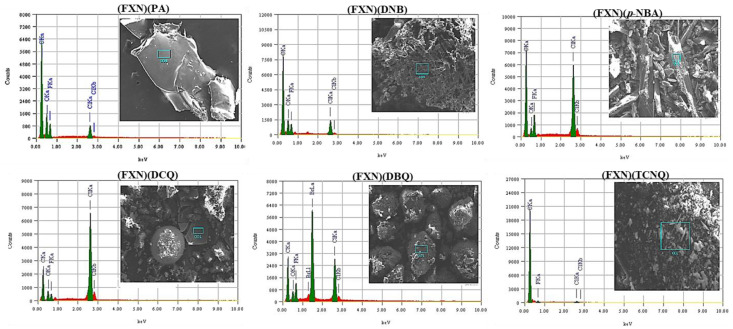
SEM images and EDX spectra of six ^1^HNMR spectra of six ligand complexes.

The form of the different (FXN)(π-acceptors) is dependent on the current acceptor according to electron micrographs due to the varying chemical compositions. EDX spectra confirm the existence of carbon, oxygen, and halogens (chlorine and fluorine) in the FXN complexes (Figure 4). The homogeneity and consistency of particle morphologies in synthesized FXN CT complexes imply that the morphological phases of the [(FXN)(π-acceptor)] complexes have a consistent matrix, with particle sizes ranging from 50 to 500 μm (Figure 4).

### 2.2. Molecular Docking Studies

Serotonin 5-HT2A (PDB ID: 6A94) and dopamine (PDB ID: 6CM4) protein receptors were docked against all synthesized CT complexes, resulting in the optimum docking pose. For comparison reasons, FXN was utilized as control. Interestingly, five of six CT complexes showed higher docking scores than that of FXN alone at both receptors (Table 1).

Table 1 shows [(FXN)(PA)] had the best docking scores out of all the [(FXN)(π-acceptor)] tested. Theoretically, [(FXN)(PA)] and serotonin have a binding energy of −9.5 kcal/mol, while dopamine has −8.8 kcal/mol. Interestingly, the [(FXN)(PA)]–serotonin (CTpS) complex interacts more strongly than dopamine due to its greater binding-energy values.

**Figure 5:**
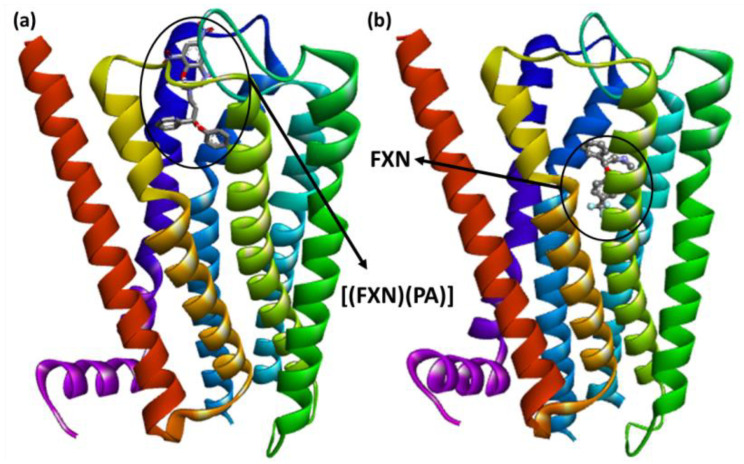
Best-docked pose showing a helical model of (**a**) [(FXN)(PA)]–serotonin; (**b**) (FXN)–serotonin.

The interaction data and best docking pose of [(FXN)(PA)]–serotonin and (FXN)–serotonin are displayed in Figure 5 and Table 2.

**Figure 6:**
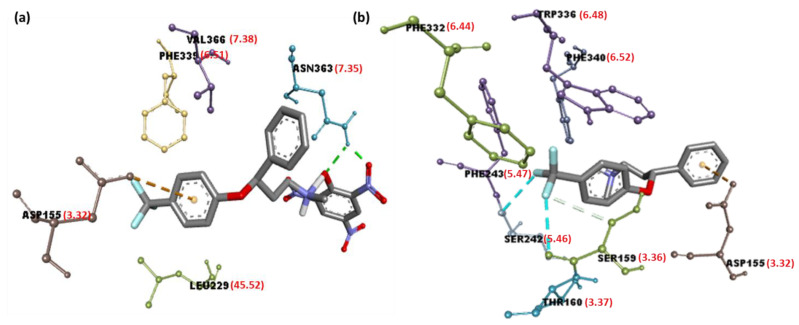
Three-dimensional illustrations of interactions of (**a**) [(FXN)(PA)]–serotonin and (**b**) (FXN)–serotonin, representing Ballesteros–Weinstein nomenclature in red brackets.

The 3D illustrations of the interactions between [(FXN)(PA)]–serotonin or (FXN)–serotonin are presented in Figure 6a,b.

**Figure 7:**
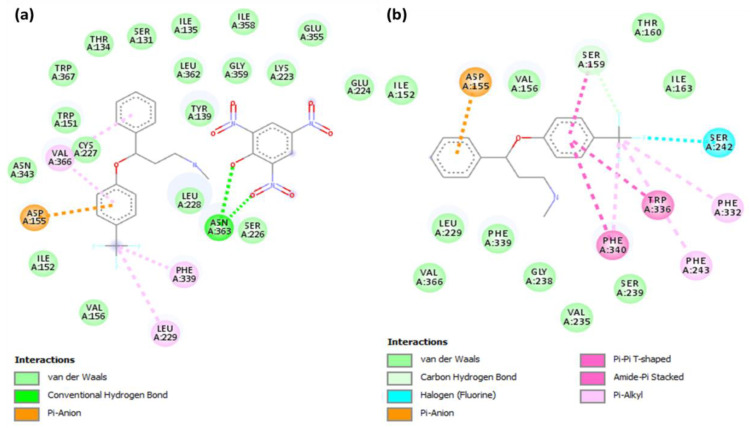
Two-dimensional illustration of interactions of (**a**) [(FXN)(PA)]–serotonin or (**b**) (FXN)–serotonin.

As shown in Figure 6a, in the [(FXN)(PA)]–serotonin complex, the amino acid residue Asn363 is responsible for the hydrogen-bond interactions. Additionally, Val366 (7.38; Ballesteros–Weinstein nomenclature), Leu229 (45.52), Phe339 (6.51) (π-Alkyl), and Asp115 (3.32) (π-Anion) interactions were present (Table 2) [33,34]. Similarly, in the [(FXN)(TCNQ)]–serotonin complex, the amino acid residue Leu80 with halogen–fluorine; and Trp367, Val364, Val84, and Ala360 with π-Alkyl interactions, can be seen. The [(FXN)(pNBA)]–serotonin complex; the amino acid residue Val251 with π-Sigma; Gly326 and Lya323 with halogen–fluorine; and Ile327 and Leu247 with π-Alkyl interactions are also present. Additionally, there are the [(FXN)(DNB)]–serotonin complex, the amino acid residue Ile152 and Asp155 with halogen–fluorine; and Val156, Val366, and Leu229 with π-Alkyl interactions. In the [(FXN)(DBQ)]–serotonin complex, the amino acid residue Asn363 with halogen–fluorine, as well as Leu80 with π-Alkyl interactions, can be seen. The other complexes with lower scores than [(FXN)(PA)] show no hydrogen bonding with serotonin receptors, as shown in Figure S1. FXN has theoretical binding energies of −8.5 and −7.9 kcal/mol when docked with serotonin and dopamine receptors, respectively. Therefore, (FXN)–serotonin (FXNS) has a larger docking score than dopamine, indicating that FXN has a stronger interaction with serotonin. On other hand, Phe243 (5.47), Phe332 (6.44) (π-Alkyl); Phe340 (6.52), Trp336 (6.48) (π-Stacked); Asp155 (3.32) (π-Anion); and Ser242 (5.46), Ser159 (3.36) (halogen–fluorine) interactions can be seen (Table 2). This indicates that [(FXN)(PA)] has the highest docking score value and binds to both receptors more effectively than the reactant donor (FXN) alone. When a substance docks or binds with a receptor, docking scores show how much energy is released. A ligand with a higher docking score, or one that is more negative, might be able to block one with a lower docking score or one that is less negative. Figure 7 displays 2D illustrations of the interactions between the ligand and receptor. Other details (name, distance, category, and type) of the interactions are illustrated in Tables S1 and S2.

### 2.3. Hydrogen Bonds, Ionizability, Hydrophobicity, and Aromatic Surfaces

**Figure 8:**
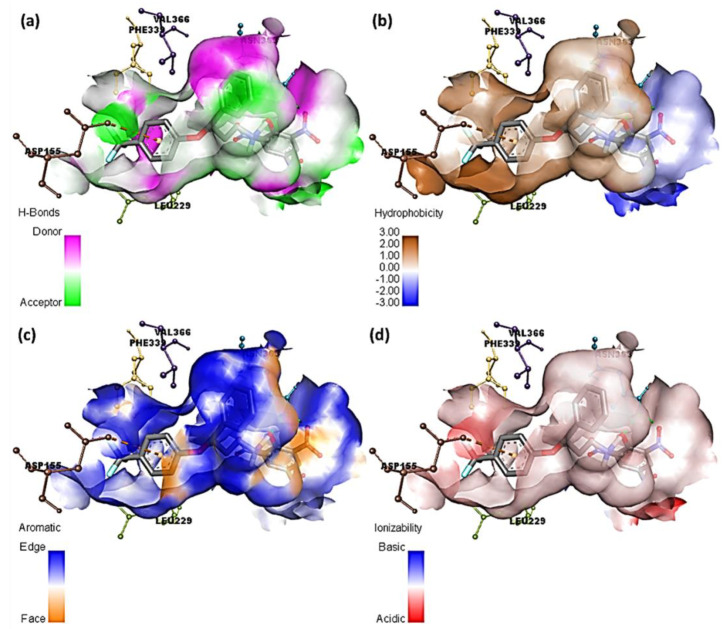
Molecular docking data of [(FXN)(PA)]–serotonin complex; (**a**) bonding surface of hydrogen bonds, (**b**) hydrophobic surfaces, (**c**) aromatic surface, and (**d**) ionizability surface.

**Figure 9:**
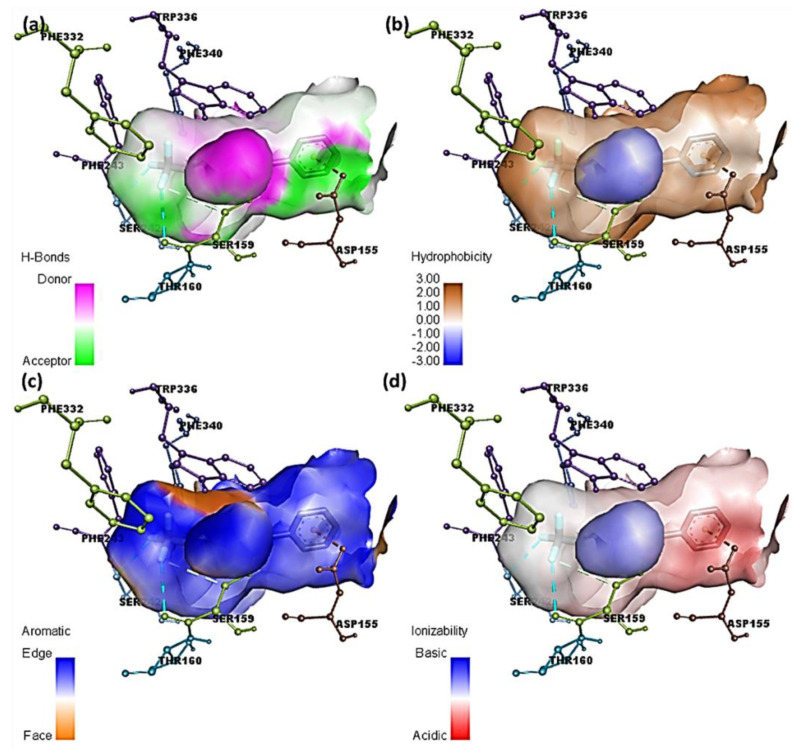
Molecular docking data of (FXN)–serotonin complex; (**a**) bonding surface of hydrogen bonds, (**b**) hydrophobic surfaces, (**c**) aromatic surface, and (**d**) ionizability surface.

The ligand–receptor complexes were assessed using DiscoveryStudio (DS) software. Mutual interactions were explored, and different surfaces were created around the ligands [35]. Figure 8 and Figure 9 display several molecular docking data at the interaction site of [(FXN)(PA)]–serotonin and (FXN)–serotonin, respectively.

Herein, fluorine atoms play a significant role in hydrogen-bond formation. The hydrogen-atom acceptor area is indicated in green, and the donor area is indicated in pink for the amino acid residues at the hydrogen-bond surface in Figure 8a and Figure S1a. The presence of the hydrophilicity features of the receptor around the ligand is confirmed by the hydrophobicity surface (Figure 8b and Figure S1b). Further, the aromatic face/edge surface (Figure 8c and Figure S1c, orange/blue = face/edge) was revealed using the docking outputs. The ionization surface reflected the acidic and basic propensity (Figure 8d and Figure S1d, blue color = basic and red color = acidic) [36].

### 2.4. MD Simulation and Structural Stability Analysis upon Ligand Binding

**Figure 10:**
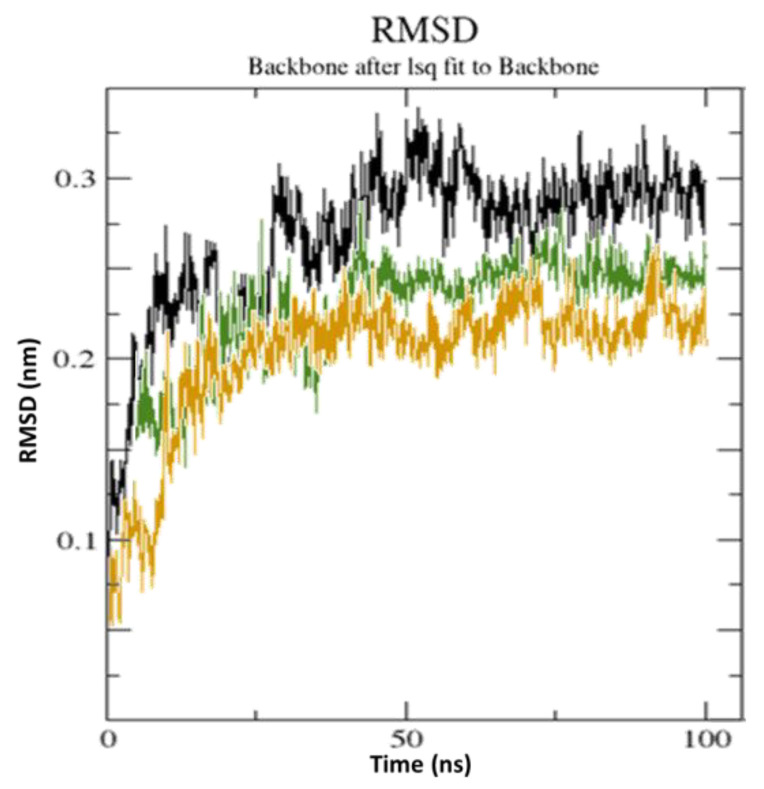
RMSD data of unbound serotonin receptor (black), (FXN)–serotonin (green), and [(FXN)(PA)]–serotonin (brown) at 100 ns.

The highest docking scores from CTpS and FXNS were used as the starting structures to achieve the MD simulation at a time of 100 ns (Figure 10). The best-docked result was used to investigate the binding process at the receptor’s active region in well-defined water environments. MD simulation data were processed for structural stability studies by determining the root mean square deviation (RMSD). As demonstrated in the RMSD graphic, both CTpS and FXNS achieved steady conformation after ~55 ns, with suitable RMSDs of 2.01 Å and 2.21 Å, respectively (Figure 10).

**Figure 11:**
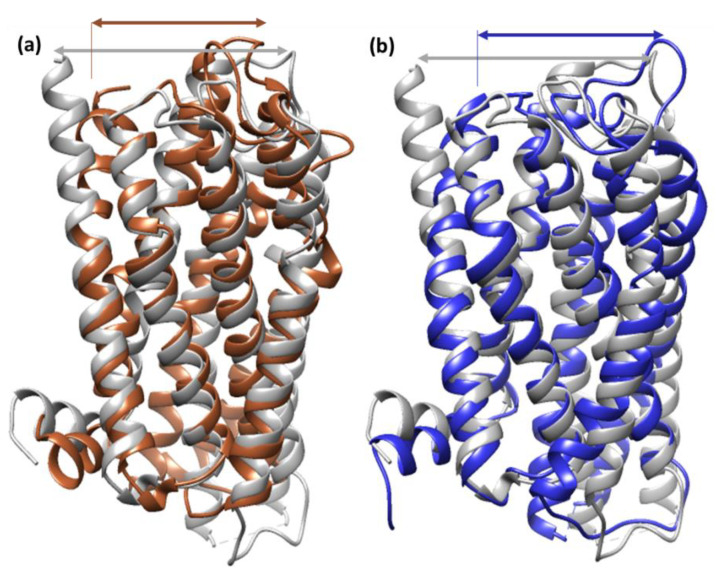
Superimposed simulation structure of (**a**) unbounded serotonin receptor (gray) and [(FXN)(PA)]–serotonin (brown); (**b**) unbounded serotonin receptor (gray) and (FXN)–serotonin (blue).

An RMSD value of <3.0 Å is widely considered to be the most acceptable and implies that the complex is suitably stable [37]. Lower RMSD values for CTpS and FXNS suggest that the ligand reduces protein flexibility, which blocks a conformational change, indicating that CTpS develops a more stable combination. The results are consistent with the theory that ligand–receptor interactions reduce the distance between protein chains and bring them closer together (Figure 11) [38].

**Figure 12:**
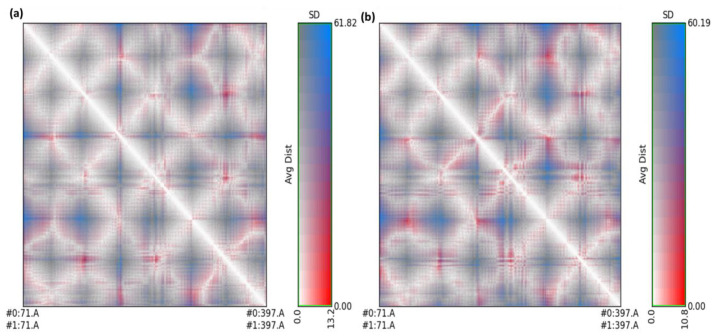
RR distance map between (**a**) unbounded serotonin and [(FXN)(PA)]–serotonin after simulation; (**b**) unbounded serotonin and (FXN)–serotonin after simulation. Average distance and standard deviation were included and calculated for all amino acid pairs.

The standard deviation and average distance between each pair of amino acids for each conformation were displayed using RR distance maps (Figure 12) [39,40].

**Figure 13:**
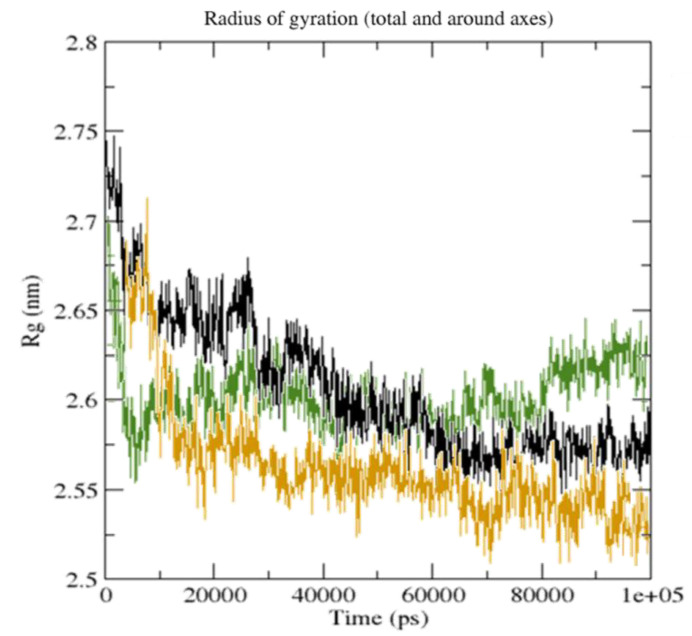
Radius of gyration of unbound serotonin alone (black), FXN–serotonin complex (green), and [(FXN)(PA)]–serotonin complex (brown) during 100 ns simulation.

The red and blue parts depict residue pairings through the highest distance differences between two amino acids, while the white diagonal represents residue pairings with a distance of zero (Figure 12). The radius of gyration (Rg) values for CTpS and FXNS were 25.62 and 26.12, respectively. The Rg for CTpS and FXNS declined during the simulation, indicating that the structures were becoming further compressed (Figure 13).

### 2.5. Hydrogen-Bond Analysis

**Figure 14:**
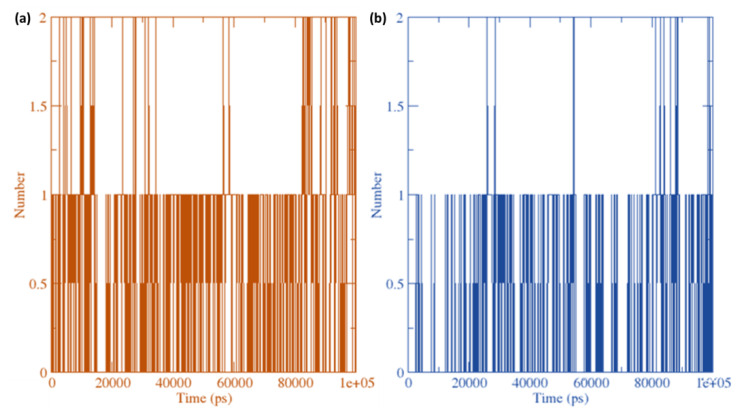
Average hydrogen bonding interactions between (**a**) [(FXN)(PA)]–serotonin and (**b**) (FXN)–serotonin at 100 ns simulation.

The amount of hydrogen-bond interactions that have arisen in ligand–receptor combinations (FXNS and CTpS) were plotted against time using a grid-search at grid = 25 × 11 × 14 and rcut = 0.35 (Figure 14).

The hydrogen bonds between ligands (FXN) or [(FXN)(PA)] with serotonin receptors were calculated (Figure 14). The average number of H-bonds per timeframe was 0.656 of 252,486 for FXN–serotonin complex and was 0.216 of 252,466 for [(FXN)(PA)]–serotonin complex. Overall, the receptor–ligand interaction was found to markedly enhance the H-bonds, with more bonds in [(FXN)(PA)]–serotonin than FXN–serotonin.

### 2.6. Solvent Accessibility Surface Area Analysis

**Figure 15:**
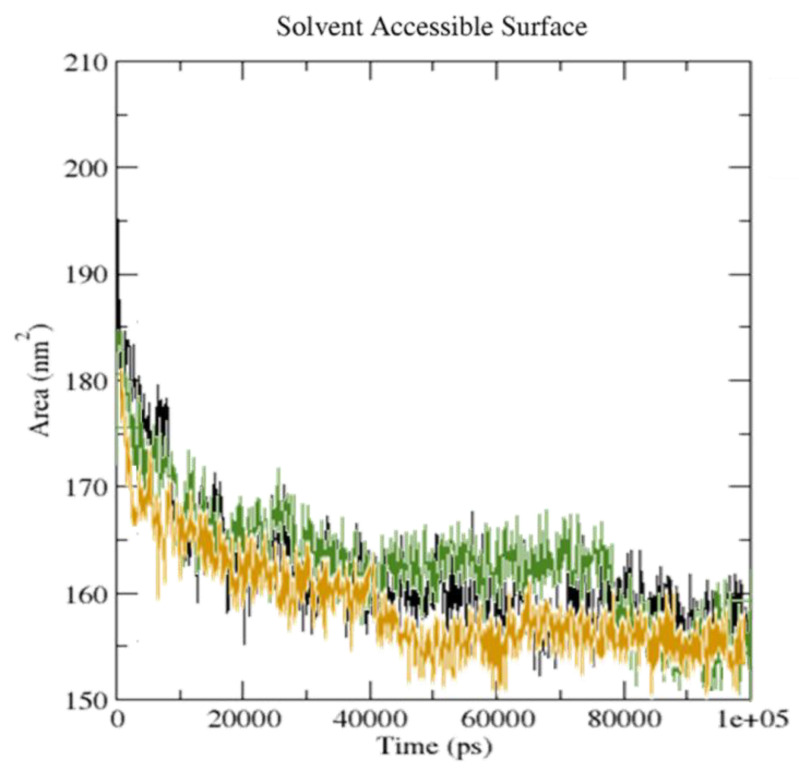
SASA analysis for unbound serotonin (black), [(FXN)(PA)]–serotonin (brown), and FXN–serotonin (green) during the 100 ns MD simulation.

The solvent accessibility surface area (SASA) was found to change because of the binding of ligand to receptor (Figure 15). 

A reduced SASA value for the serotonin upon binding to [(FXN)(PA)] suggests an adjustment in the configuration of the protein structure and a decrease in pocket size, with increased hydrophobicity around it [41].

### 2.7. DFT Studies

The CT complexes [(FXN)(PA)], [(FXN)(DCQ)], [(FXN)(DBQ)], [(FXN)(DNB)], [(FXN)(pNBA)], and [(FXN)(TCNQ)] were optimized using the B3LYP/6-311G++ level theory, and their binding energies were calculated and obtained as −637.49, −18.38, −25.01, −104.91, −87.31, and −332.74 kcal/mol, respectively.

**Figure 16:**
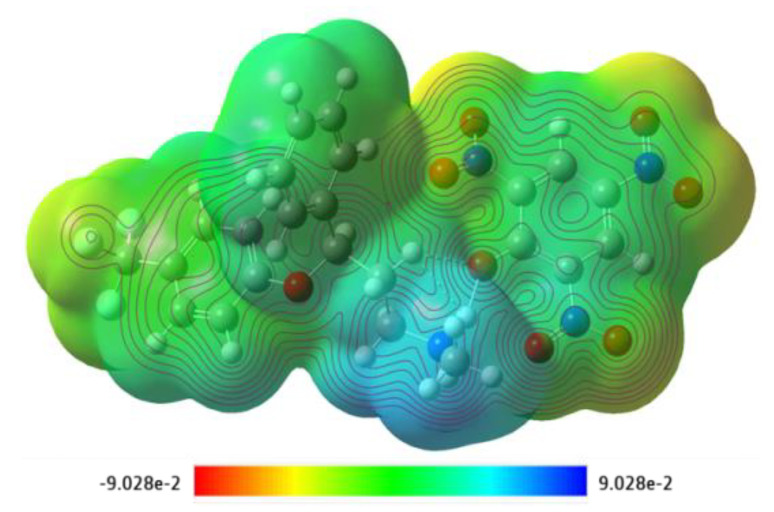
Molecular electrostatic potential surface map of [(FXN)(PA)] complex with respective color scales.

The optimized geometry of CT complexes [(FXN)(PA)], [(FXN)(DCQ)], [(FXN)(DBQ)], [(FXN)(DNB)], [(FXN)(pNBA)], and [(FXN)(TCNQ)] with Mulliken at-atomic coordinates and strain-free lattice constants is presented in Figure S1. The obtained bond lengths from the optimized structure of [(FXN)(PA)] are shown in Figure S2. Bond angles and lengths are presented in Tables S3 and S4. Mulliken charges of the complex were also obtained and are provided in Table S5. The strength of the electrostatic potentials of [(FXN)(PA)] is depicted in molecular electrostatic potential (MEP) surface map (Figure 16).

Blue represents the electropositive region, while red represents electronegative areas. These findings point to the molecule’s preferred binding sites for electrophilic and nucleophilic charges [42]. The MEP surface is mapped with the color scale from deep red (−9.028 × 10^−2^) to deep blue (+9.028 × 10^−2^) (Figure 16) [43].

**Figure 17:**
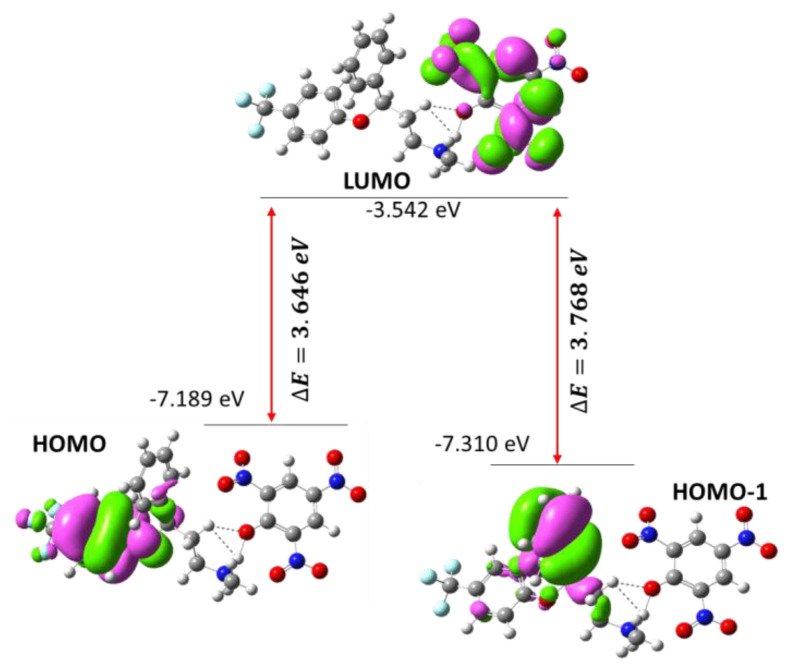
The energy gaps and spatial plot of HOMO and LUMO.

TD-DFT was used to examine the nature of electronic transitions in water. The TD-DFT yielded two electronic absorption bands at 436 and 451 nm. HOMO → LUMO and HOMO-1 → LUMO were allocated at 451 and 436 nm, respectively. The electron acceptors were mainly LUMO and electron donors were HOMO, as observed in the FXN moiety of the CT complex [(FXN)(PA)]. Figure 17 represents the HOMO and LUMO spatial arrangements and their gaps and associated energies.

**Figure 18:**
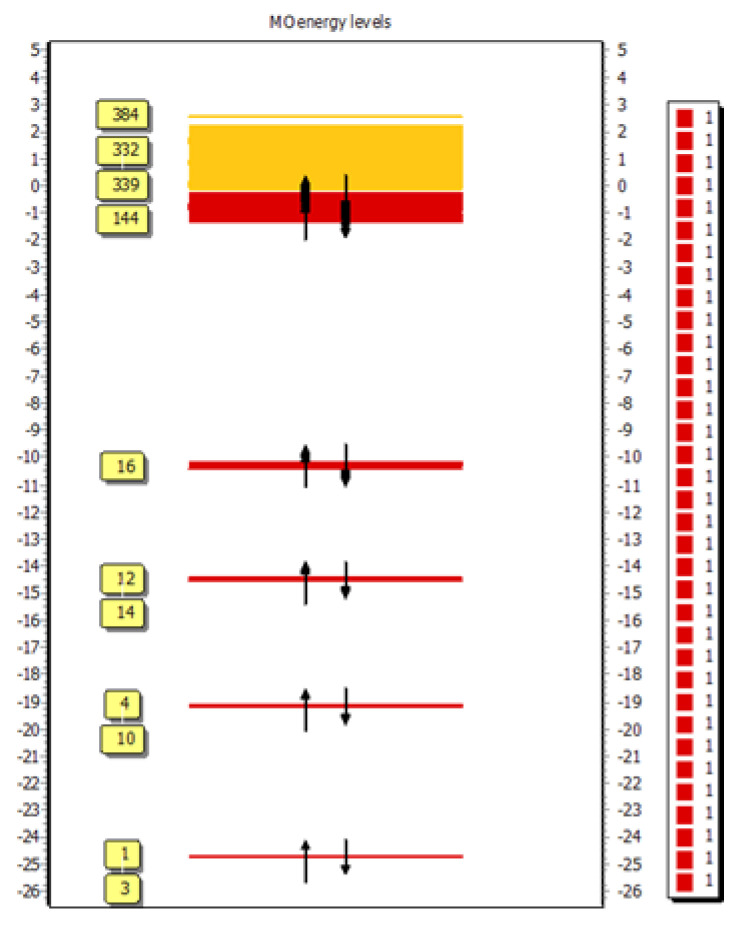
MO energy-level diagram of the CT complex [(FXN)(PA)].

The molecular orbital energy level diagram of the CT complex [(FXN)(PA)] is presented in Figure 18.

The HOMO–LUMO and HOMO-1–LUMO gaps (∆E) for [(FXN)(PA)] were 3.646 and 3.768 eV, respectively [44]. Based on these DFT results, some theoretical molecular parameters related to chemical reactivity in water as solvent are presented in Table 3 [45,46].

**Figure 19:**
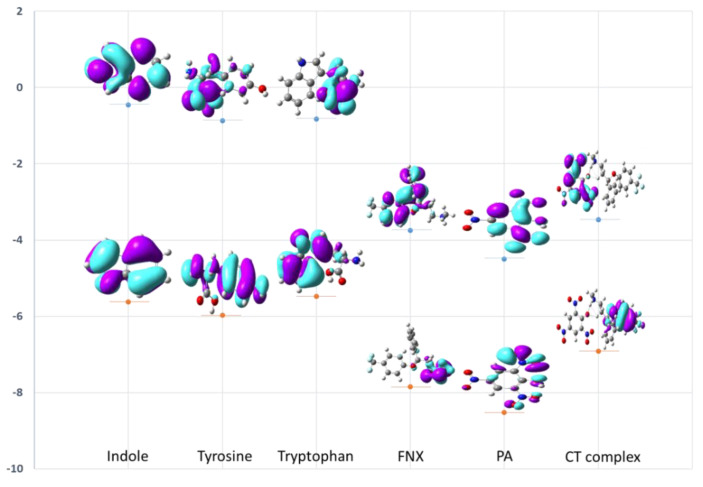
HOMO and LUMO energy levels of the molecular orbitals for indole, tyrosine, tryptophan, FXN, PA, and [(FXN)(PA)].

DFT calculations were employed to explain the stability of the CT complex [(FXN)(PA)] (Figure 19). To study the effect of the aromatic residues (indole, tyrosine, and tryptophan) of the protein on the CT complex [(FXN)(PA)], the HOMO LUMO energy and band gap were calculated for indole, tyrosine, tryptophan, FXN, PA, and [(FXN)(PA)] (Figure 19). The energy difference between HOMO and LUMO has an impact on how chemically stable molecules are. As previously reported, ligands with a smaller band gap are soft in nature, have low kinetic stability, and higher chemical reactivity [47]. The opposite is true for ligands with a wide energy gap, which are harder by nature, more unstable, and more chemically reactive [45,47]. The band gap energy of [(FXN)(PA)] is calculated as 3.646 eV, which is the smallest among all of the complexes, suggesting higher stability. On the other hand, the obtained energy of LUMO = −4.494 eV and HOMO = −8.524 eV for PA, and LUMO = −3.746 eV and HOMO = −7.849 eV for FXN (Table 4), validates the easy transfer of electrons from HOMO of FXN to LUMO of PA, and results in the formation of a stable CT complex with a smaller band gap [(FXN)(PA)]. Furthermore, the calculated LUMO energies of the aromatic residues (indole, tyrosine, and tryptophan) of the protein were not in the sequence (close enough) to dissociate the charge transfer bond between FXN and PA, ensuring the stability of the [(FXN)(PA)] complex [42,44,48,49].

## 3. Materials and Methods

### 3.1. Preface 

**Figure 20:**
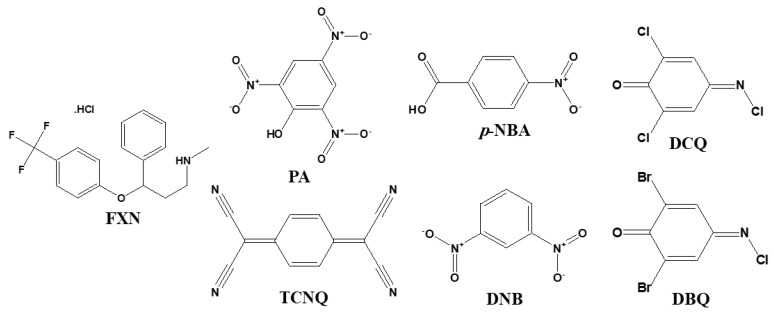
Structures of fluoxetin HCl (FXN) and six π-acceptors.

We purchased all chemicals from Aldrich and Fluka Chemicals. We utilized FXN drug and the six π-acceptors without additional purification: picric acid (PA); p-nitro benzoic acid (p-NBA); 2,6-dichloroquinone-4-chloroimide (DCQ); 2,6-dibromoquinone-4-chloroimide (DBQ); dinitrobenzene (DNB); and 7,7′,8,8′-tetracyanoquinodimethan (TCNQ) (Figure 20).

In order to make the solid CT complexes, we mixed 0.309 gm (1 mmol) fluoxetin hydrochloride with 1 mmol of each π-acceptor in 20 mL of chloroform solvent [50].

At room temperature, we mixed all of the combinations for 1 h. We used filtering to remove the solid products, subsequently washed the sample with minimal volumes of chloroform, then dehydrated under vacuum with CaCl_2_.

### 3.2. Molecular Docking

We used OpenBabelIGUI software version 2.4.1 [51] to obtain the structure of FXN and the six CT complexes in PDBQT format. We used MMFF94 force field and conjugate gradient optimization algorithm to diminish the energy of the structures [52]. We used the conjugate gradient optimization algorithm using PyRx-Python prescription 0.8 for 500 steps. We employed the RCSB protein data repository to obtain the 3D crystal structures of serotonin 5-HT2A (PDB ID: 6A94) and dopamine (PDB ID: 6CM4) [53]. To add polar hydrogen atoms to the receptors and determine their Kollman charges, we utilized the Autodock Tool [54]. To assign partial charges, we employed the Geistenger approach. We used Autodock Vina to carry out the docking calculations [55]. We used a grid of 54 × 40 × 42 and center x = 13.562, center y = 0.255, center z = 61.097 with an exhaustiveness value of 8. We selected the docked positions on the basis of good docking scores and examined the interactions using DS Visualizer.

### 3.3. MD Simulation Study

**Figure 21:**
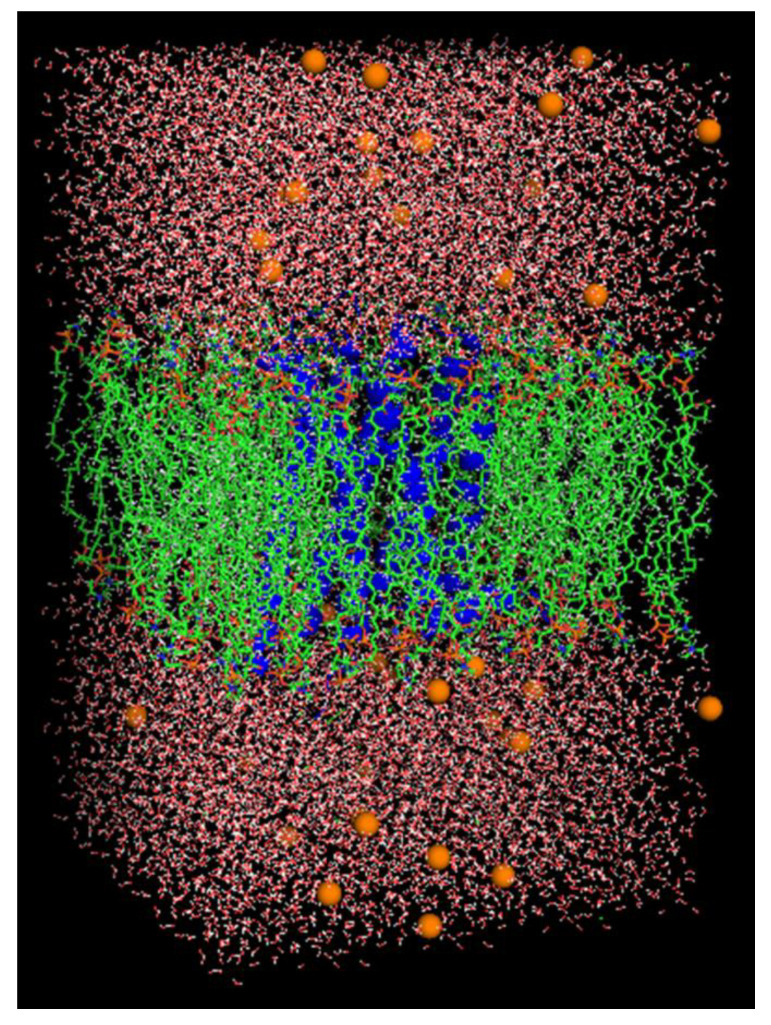
Lateral view of [(FXN)(PA)] complex incorporated in dipalmitoylphosphatidylcholine (DPPC) membrane in rectangular box solvated with water molecules and neutralized with 52 K^+^ and 57 Cl^−^ ions (0.15 M salt).

We accomplished the MD simulation using the best-docked complexes of the receptor and ligand with a high docking score for FXN alone and [(FXN)(PA)]. We attained the topologies and parameters of the ligands by CGenFF with CHARMM-GUI [56,57]. We used online server CHARMM-gui to insert the DPPC (dipalmitoylphosphatidylcholine) membrane. We added lower- and upper leaflet with 72 DPPC molecules. We used SPC water models that extended 10 Å from the receptor to examine the receptor–ligand configurations in a rectangular box [58]. We administered 52 K^+^ and 57 Cl^−^ ions (0.15 M salt) to neutralize the systems and reproduce physiological salt concentrations (Figure 21). In the NPT/NVT equilibration run, we subjected both systems to periodic boundary conditions at constant temperature (300 K) and pressure (1.0 bar) using Leap-frog MD integrator for 100 ns simulation time [59].

To remove inappropriate links inside the system, we reduced energy using the steepest descent approach with 5000 steps [60]. We employed the GROMACS program to accomplish the trajectory investigation [61].

We assessed the root mean square deviation by gmx rms tool. We studied hydrogen bonding by Gmxhbond instrument. We used the gmx gyrate and gmxsasa tools to calculate the gyration radius and solvent-accessible surface area. We created plots with Grace Software and created pictures with Polo/VMD [62,63].

### 3.4. Density Functional Theory

We performed DFT analysis by Gaussian 09RevD.01 package [64]. We used the calculations to obtain a stable molecular geometry and study the electronic transitions in the CT complex [(FXN)(PA)] from a theoretical perspective. Furthermore, we applied B3LYP/6-311G++ basic to obtain the optimized structure of the CT complexes [65]. Then, we assessed the electrostatic potential map (MEP), LUMO, and HOMO spatial plots of the [(FXN)(PA)] complex [66]. The system’s chemical stability is partly determined by the boundary molecular orbitals. We also investigated several structure-based molecular properties. We also used this theory to calculate structure-based molecular parameters, such as bond lengths, angles, atomic charges, total energy, electronic properties, and protein stability. We used ChemCraft 1.5 software for visualization [67].

## 4. Conclusions

We evaluated the 1:1 complexes of FXN with six π-acceptor complexes in both solid and liquid phases. Several spectroscopic analyses were used to characterize the isolated complexes. Additionally, molecular docking demonstrated that [(FXN)(PA)] has the maximum immunomolecular docking score towards the serotonin [5-HT2A (PDB ID: 6A94)] receptor, and was more effective than the FXN reactant donor alone. It was also found that at 100 ns MD simulation, [(FXN)(PA)]–serotonin was more stable than (FXN)–serotonin. Furthermore, the theoretical data from the DFT calculations helped to explore the molecular geometry of the CT complexes along with their important physical parameters. The stability of the CT complex [(FXN)(PA)] was also studied by DFT calculations.

## Figures and Tables

**Table 1:** Docking score of all [(FXN)(π-acceptor)] complexes and FXN drug alone into two protein receptors [serotonin (PDB ID: 6A94) and dopamine (PDB ID: 6CM4)].

Ligands	Docking Score (kcal/mol)	
	PDB ID: 6A94	PDB ID: 6CM4
FXN-TCNQ	−7.8	−7.4
FXN-*p*NBA	−6.2	−5.9
FXN-DNB	−7.6	−7.1
FXN-DBQ	−8.1	−7.9
FXN-DCQ	−7.8	−7.2
FXN-PA	−9.5	−8.8
FXN	−8.5	−7.9

**Table 2:** Interaction data of [(FXN)(PA)]–serotonin and (FXN)–serotonin (PDB ID: 6A94).

Ligands	Docking Score (kcal/mol)	Interactions	
		H-Bond	Others
[(FXN)(PA)]–serotonin	−9.5	Asn363	Val7.38, Leu45.52, Phe6.51(π-Alkyl); Asp3.32(π-Anion)
(FXN)–serotonin	−8.5		Phe5.47, Phe6.44(π-Alkyl); Phe6.52, Trp6.48 (π-Stacked); Asp3.32(π-Anion); Ser5.46, Ser3.36 (Halogen-Fluorine)

**Table 3:** Theoretical molecular characteristics and components of CT complex [(FXN)(PA)].

Parameters.	RB3LYP/6-311G++
Minimum SCF energy (a.u.)	−2008.551032
Polarizability (α) (a.u.)	302.014352
Dipole Moment (Debye)	9.730154
Zero-point vibrational energy (kcal/mol)	248.24365
Total thermal energy (kcal/mol)	251.492
Electronic spatial extent (a.u.)	29,874.6801
Frontier MO energies (eV)	
LUMO	−3.542
HOMO	−7.189
HOMO-1	−7.310
Gap (LUMO–HOMO)	3.646
Gap (LUMO–HOMO-1)	3.768

**Table 4:** HOMO and LUMO energies calculated for tyrosine, tryptophan, FXN, PA, and [(FXN)(PA)] at B3LYP/6-311G++ levels of theory.

	Indole	Tyrosine	Tryptophan	FXN	PA	[(FXN)(PA)]
LUMO (eV)	−0.456	−0.872	−0.827	−3.746	−4.494	−3.542
HOMO (eV)	−5.617	−5.974	−5.451	−7.847	−8.524	−7.189
Band gap (eV)	5.160	5.102	4.653	4.101	4.029	3.646

## Data Availability

Data is contained within article.

## Supplementary Materials

The following supporting information can be downloaded at: https://www.mdpi.com/article/10.3390/molecules27185883/s1: Figure S1. 2D illustration of the interactions of (a) [(FXN)(TCNQ)]-serotonin, (b) [(FXN)(pNBA)]-serotonin, (c) [(FXN)(DNB)]-serotonin, and (d) [(FXN)(DCQ)]-serotonin. Figure S2: Optimized structure of the CT complexes—(a) [(FXN)(PA)], (b) [(FXN)(DCQ)], (c) [(FXN)(DBQ)], (d) [(FXN)(DNB)], (e) [(FXN)(pNBA)], and (f) [(FXN)(TCNQ)] with Mulliken atom-numbering scheme. Figure S3: Optimized structure of the CT complex [(FXN)(PA)] showing bond lengths. Table S1: [(FXN)(PA]–serotonin interaction results by DS. Table S2: FXN–serotonin interaction results by DS. Table S3: The bond lengths of the CT complex [(FXN)(PA)] obtained through DFT. Table S4: The bond angles of the CT complex [(FXN)(PA)] obtained through DFT. Table S5: Mulliken atomic charges of the CT complex [(FXN)(PA)] atoms.
