# Supplementary material for: Multispectral and Molecular Docking Studies Reveal Potential Effectiveness of Antidepressant Fluoxetine by Forming π-Acceptor Complexes
Source: Molecules. 2022 Sep 10;27(18):5883. doi: 10.3390/molecules27185883 (PMC9505585; doi:10.3390/molecules27185883)
Supplement: Supplementary file 1 [file molecules-27-05883-s001.zip › molecules-1875183-supplementary.pdf]

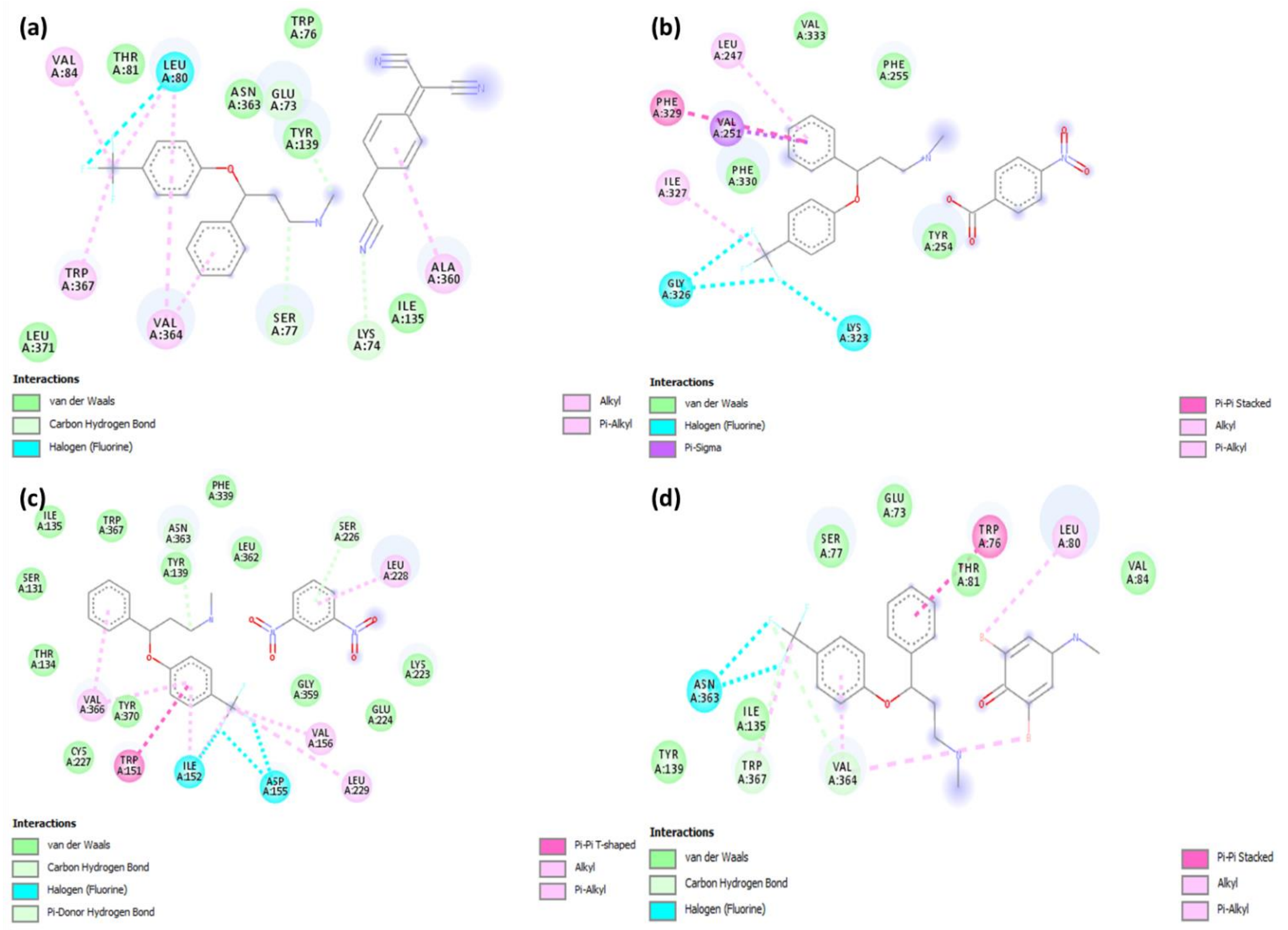

Figure S1. 2D illustration of the interactions of (a) [(FXN)(TCNQ)]-serotonin, (b) [(FXN)(pNBA)]-serotonin, (c) [(FXN)(DNB)]-serotonin, and (d) [(FXN)(DCQ)]-serotonin**.**

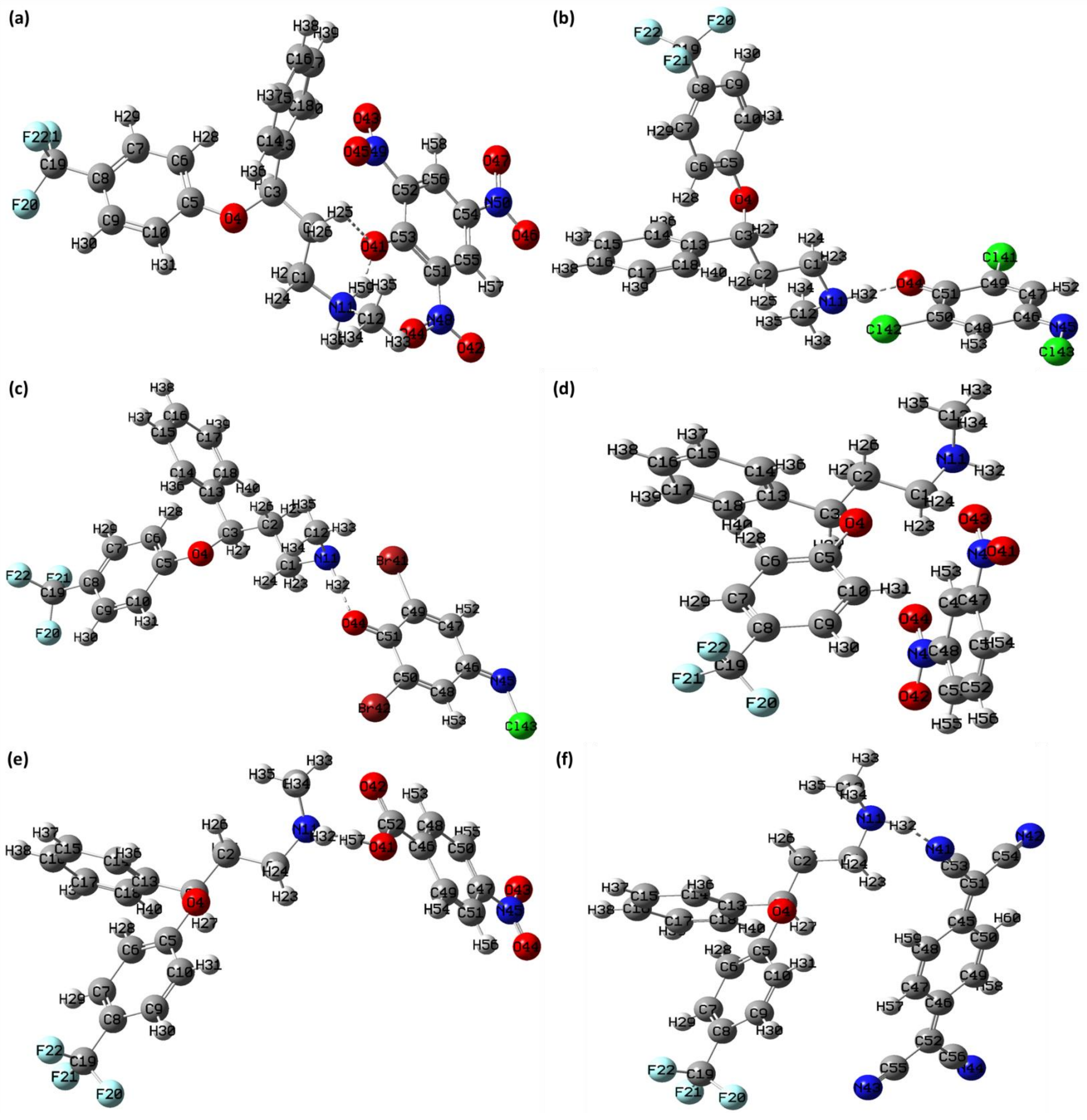

Figure S2. Optimized structure of the CTcomplexes- (a)[(FNX)(PA)], (b) [(FNX)(DCQ)], (c) [(FNX)(DBQ)], (d) [(FNX)(DNB)], (e) [(FNX)(pNBA)], and (f) [(FNX)(TCNQ)] with Mulliken atom numbering scheme.

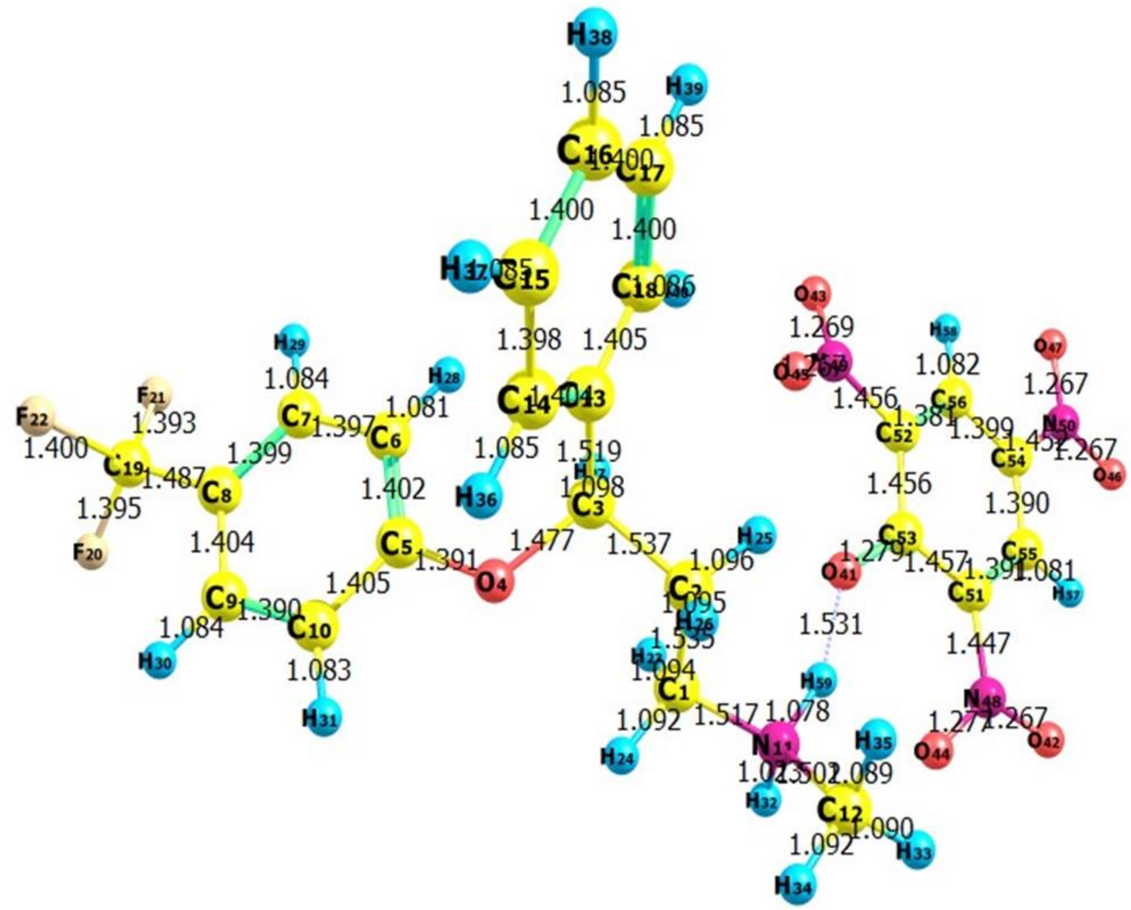

Figure S3. Optimized structure of the CT complex [(FNX)(PA)] showing bond lengths.

Table S1. [(FXN)(PA]-serotonin interactions results by DS.

Name	Distance	Category	Type
ASN363:H - [(FXN)(PA]:O	2.29436	Hydrogen Bond	Conventional Hydrogen Bond
ASN363:H - [(FXN)(PA]:O	1.9154	Hydrogen Bond	Conventional Hydrogen Bond
ASP155:O - [(FXN)(PA]	4.54706	Electrostatic	Pi-Anion
[(FXN)(PA]:C - LEU229	4.75129	Hydrophobic	Alkyl
PHE339 - [(FXN)(PA]:C	4.99763	Hydrophobic	Pi-Alkyl
[(FXN)(PA] - VAL366	5.06449	Hydrophobic	Pi-Alkyl
[(FXN)(PA] - VAL366	4.80608	Hydrophobic	Pi-Alkyl

TableS2. FXN-serotonin interactions results by DS.

Name	Distance	Category	Type
SER159:C - FXN:F	3.46786	Hydrogen Bond	Carbon Hydrogen Bond
SER159:O - FXN:F1	3.05844	Halogen	Halogen (Fluorine)
SER242:O - :FXN:F2	3.05139	Halogen	Halogen (Fluorine)
ASP155:O - FXN	3.50266	Electrostatic	Pi-Anion
TRP336 - FXN	4.81774	Hydrophobic	Pi-Pi T-shaped
PHE340 - FXN	4.91544	Hydrophobic	Pi-Pi T-shaped
SER159:C,O;THR160:N - FXN	5.10599	Hydrophobic	Amide-Pi Stacked
PHE243 - FXN:C17	5.39225	Hydrophobic	Pi-Alkyl
PHE332 - FXN:C17	5.12623	Hydrophobic	Pi-Alkyl
PHE340 - FXN:C17	4.80155	Hydrophobic	Pi-Alkyl

Table S3. The bond lengths of the CT complex [(FNX)(PA)] obtained through DFT.

S.No.	[(FNX)(PA)](RB3LYP/6-311G++)			
	AtomNo.	Bondlength(Å)	AtomNo.	Bondlength(Å)
**1**	R(1-2)	1.535	R(14-36)	1.085
**2**	R(1-11)	1.517	R(15-16)	1.4
**3**	R(1-23)	1.094	R(15-37)	1.085
**4**	R(1-24)	1.092	R(16-17)	1.4
**5**	R(2-3)	1.537	R(16-38)	1.085
**6**	R(2-25)	1.096	R(17-18)	1.4
**7**	R(2-26)	1.095	R(17-39)	1.085
**8**	R(3-4)	1.477	R(18-40)	1.086
**9**	R(3-13)	1.519	R(19-20)	1.395
**10**	R(3-27)	1.098	R(19-21)	1.393
**11**	R(4-5)	1.391	R(19-22)	1.4
**12**	R(5-6)	1.402	R(41-53)	1.279
**13**	R(5-10)	1.405	R(42-48)	1.267
**14**	R(6-7)	1.397	R(43-49)	1.269
**15**	R(6-28)	1.081	R(44-48)	1.277
**16**	R(7-8)	1.399	R(45-49)	1.267
**17**	R(7-29)	1.084	R(46-50)	1.267
**18**	R(8-9)	1.404	R(47-50)	1.267
**19**	R(8-19)	1.487	R(48-51)	1.447
**20**	R(9-10)	1.39	R(49-52)	1.456
**21**	R(9-30)	1.084	R(50-54)	1.452
**22**	R(10-31)	1.083	R(51-53)	1.457
**23**	R(11-12)	1.502	R(51-55)	1.391
**24**	R(11-32)	1.023	R(52-53)	1.456
**25**	R(11-59)	1.078	R(52-56)	1.381
**26**	R(12-33)	1.09	R(54-55)	1.39
**27**	R(12-34)	1.092	R(54-56)	1.399
**28**	R(12-35)	1.089	R(55-57)	1.081
**29**	R(13-14)	1.404	R(56-58)	1.082
**30**	R(13-18)	1.405	R(41-59)	1.531
**31**	R(14-15)	1.398		

Table S4. The bond angles of the CT complex [(FNX)(PA)] obtained through DFT.

S.No.	[(FNX)(PA)](RB3LYP/6-311G++)			
	AtomNo.	BondAngle(Å)	AtomNo.	BondAngle(Å)
**1**	A(2-1-11)	111.8	A(11-59-41)	157.9
**2**	A(2-1-23)	110.4	A(33-12-34)	110.8
**3**	A(2-1-24)	111.5	A(33-12-35)	109.4
**4**	A(1-2-3)	111.7	A(34-12-35)	109.9
**5**	A(1-2-25)	109.7	A(14-13-18)	119.6
**6**	A(1-2-26)	110.8	A(13-14-15)	120.2
**7**	A(11-1-23)	105.5	A(13-14-36)	119.5
**8**	A(11-1-24)	108.7	A(13-18-17)	120
**9**	A(1-11-12)	116	A(13-18-40)	120
**10**	A(1-11-32)	109.7	A(15-14-36)	120.3
**11**	A(1-11-59)	107	A(14-15-16)	120.2
**12**	A(23-1-24)	108.7	A(14-15-37)	119.8
**13**	A(3-2-25)	107	A(16-15-37)	120.1
**14**	A(3-2-26)	108.6	A(15-16-17)	119.8
**15**	A(2-3-4)	104.8	A(15-16-38)	120.1
**16**	A(2-3-13)	112.4	A(17-16-38)	120.1
**17**	A(2-3-27)	108.7	A(16-17-18)	120.3
**18**	A(25-2-26)	108.9	A(16-17-39)	120.1
**19**	A(4-3-13)	112.2	A(18-17-39)	119.6
**20**	A(4-3-27)	108.3	A(17-18-40)	120
**21**	A(3-4-5)	120.7	A(20-19-21)	107.2
**22**	A(13-3-27)	110.2	A(20-19-22)	105.7
**23**	A(3-13-14)	120.7	A(21-19-22)	106
**24**	A(3-13-18)	119.7	A(41-53-51)	124.8
**25**	A(4-5-6)	124.5	A(41-53-52)	121.5
**26**	A(4-5-10)	115.2	A(53-41-59)	138.7
**27**	A(6-5-10)	120.4	A(42-48-44)	121.5
**28**	A(5-6-7)	119.3	A(42-48-51)	118.5
**29**	A(5-6-28)	121.3	A(43-49-45)	122.8
**30**	A(5-10-9)	120	A(43-49-52)	117.6
**31**	A(5-10-31)	118.8	A(44-48-51)	120
**32**	A(7-6-28)	119.4	A(45-49-52)	119.6
**33**	A(6-7-8)	120.5	A(46-50-47)	124
**34**	A(6-7-29)	119.6	A(46-50-54)	118.1
**35**	A(8-7-29)	119.9	A(47-50-54)	117.9
**36**	A(7-8-9)	119.8	A(48-51-53)	121.4
**37**	A(7-8-19)	120.3	A(48-51-55)	116
**38**	A(9-8-19)	119.8	A(49-52-53)	120.5
**39**	A(8-9-10)	120	A(49-52-56)	116.3
**40**	A(8-9-30)	119.9	A(50-54-55)	119.4
**41**	A(8-19-20)	112.2	A(50-54-56)	119.4
**42**	A(8-19-21)	112.5	A(53-51-55)	122.6
**43**	A(8-19-22)	112.9	A(51-53-52)	113.8
**44**	A(10-9-30)	120	A(51-55-54)	119.8
**45**	A(9-10-31)	121.3	A(51-55-57)	119.9
**46**	A(12-11-32)	108.7	A(53-52-56)	123.2

**47**	A(12-11-59)	109.7	A(52-56-54)	119.4
**48**	A(11-12-33)	107.8	A(52-56-58)	120.2
**49**	A(11-12-34)	109.9	A(55-54-56)	121.2
**50**	A(11-12-35)	108.9	A(54-55-57)	120.3
**51**	A(32-11-59)	105.2	A(54-56-58)	120.4

Table S5. Mulliken atomic charges of the CT complex [(FNX)(PA)] atoms.

**S.No.**	**Synthesizedcomplex**			
	**Mullikenatomic numbers**	**Mullikenatomiccharges**	**Mullikenatomic numbers**	**Mullikenatomiccharges**
**1**	1C	-0.11393	31H	0.1429
**2**	2C	-0.27234	32H	0.37164
**3**	3C	0.01759	33H	0.2177
**4**	4O	-0.59644	34H	0.17726
**5**	5C	0.30873	35H	0.20164
**6**	6C	-0.12734	36H	0.14117
**7**	7C	-0.12458	37H	0.13073
**8**	8C	-0.01528	38H	0.13209
**9**	9C	-0.10953	39H	0.13799
**10**	10C	-0.14373	40H	0.17739
**11**	11N	-0.68421	41O	-0.56844
**12**	12C	-0.28452	42O	-0.28615
**13**	13C	0.06085	43O	-0.29039
**14**	14C	-0.13773	44O	-0.36246
**15**	15C	-0.13076	45O	-0.2871
**16**	16C	-0.11455	46O	-0.29116
**17**	17C	-0.13805	47O	-0.29029
**18**	18C	-0.15477	48N	0.0416
**19**	19C	0.68878	49N	0.04014
**20**	20F	-0.28482	50N	0.03257
**21**	21F	-0.28338	51C	0.29762
**22**	22F	-0.27489	52C	0.27222
**23**	23H	0.18708	53C	0.33923
**24**	24H	0.18534	54C	0.29149
**25**	25H	0.21882	55C	-0.11157
**26**	26H	0.15191	56C	-0.08503
**27**	27H	0.16172	57H	0.23793
**28**	28H	0.16271	58H	0.23549
**29**	29H	0.16952	59H	0.46715
**30**	30H	0.16439		
